# Preparation and Evaluation of Liposomes Co-Loaded with Doxorubicin, Phospholipase D Inhibitor 5-Fluoro-2-Indolyl Deschlorohalopemide (FIPI) and D-Alpha Tocopheryl Acid Succinate (α-TOS) for Anti-Metastasis

**DOI:** 10.1186/s11671-019-2964-4

**Published:** 2019-04-18

**Authors:** Maoyuan Song, Jiaxing Wang, Jiongxi Lei, Guanghua Peng, Wenxi Zhang, Yuanyuan Zhang, Mengya Yin, Jiajia Li, Yajie Liu, Xiaomeng Wei, Xinru Li, Guiling Li

**Affiliations:** 1 Institute of Medicinal Biotechnology, Chinese Academy of Medical Science and Peking Union Medical College, Beijing, 100050 China; 2 Beijing Key Laboratory of Molecular Pharmaceutics and New Drug System, School of Pharmaceutical Sciences, Peking University Health Science Center, Beijing, 100191 China

**Keywords:** FIPI, DOX, α-TOS, Liposomes, Anti-metastasis

## Abstract

Tumor metastasis has become a key obstacle to cancer treatment, which causes high mortality. Nowadays, it involves multiple complex pathways, and conventional treatments are not effective due to fewer targets. The aims of the present study were to construct a novel liposome delivery system co-loading a specific PLD inhibitor 5-fluoro-2-indolyldes-chlorohalopemide (FIPI) in combination with antitumor drug doxorubicin (DOX) and functional excipient D-alpha tocopheryl acid succinate (α-TOS) for anti-metastasis. In this study, the liposomes containing three components (DFT-Lip) with different physicochemical properties were successfully prepared by film dispersion method combined with pH-gradient method. Physicochemical parameters such as particles size, potential, encapsulation efficiency, stability, and release profiles were investigated. In vitro and in vivo anti-metastasis effectiveness against highly metastatic breast cancer MDA-MB-231 cell line was evaluated. The liposomes showed uniform particle size (approximately 119 nm), high drug encapsulation efficiency (> 90%), slow release characteristics and stability. In vitro anti-tumor cell metastasis study demonstrated DFT-Lip could greatly inhibit motility, migration and invasion of MDA-MB-231 cells compared to other liposomes, predicting a synergistic anti-tumor metastasis effect between FIPI with α-TOS in liposomes. In vivo anti-metastasis study showed that DFT-Lip prevented the initiation and the progression of metastasis of high metastatic breast cancer. These results suggested that the liposomes containing DOX, FIPI, and α-TOS might be a promising strategy for metastatic tumor therapy in clinics.

## Introduction

Metastasis, an important hallmark of malignant tumors [1], is defined as the spread of malignant cells from the primary tumor to one or more other discontiguous organs [2]. Although surgery, radiation therapy, and chemotherapy have been relatively successful in controlling the primary tumor, the cancer remained incurable if tumor metastasis occurred [3, 4]. Tumor metastasis, which usually predicted poor prognosis and inevitable death, remained a great challenge in the clinical treatment of cancer.

The key to the prevention, delay, and treatment of tumor metastasis is to understand its mechanisms. Many studies have focused on complex biochemical processes of metastasis during the past few decades. These researchers divided metastasis into a series of sequential and interrelated steps [5–7], each of which could block the formation of metastatic lesions by being inhibited [8]. The potential therapeutic targets included epithelial-mesenchymal transition (EMT) process [9], mesenchymal-epithelial transition (MET) process [10], cancer stem cells (CSCs) [11], circulating tumor cells (CTCs) [6], disseminated tumor cells (DTCs) [6], and cell mobility [12].

Phospholipase D (PLD) enzymes as important members of phospholipase superfamily are present in a broad range of organisms such as viruses, yeast, bacteria, animals, and plants [13, 14]. Mammalian cells encode two classic PLD isoforms, PLD1, and PLD2 [15]. Evaluated PLD activity, as well as expression, has been reported in a variety of cancers [16, 17]. Although exact pathways and mechanisms were still unclear, PLDs have been proposed to play multiple cell biological roles such as the formation of lamellipodia [18], migration [17], cellular movement [19], invasion, and metastasis [20] in cancer through several molecular mechanisms. Given the fact that PLD played a crucial role in the invasion and metastasis of tumor cells, it can be speculated that inhibition of phospholipase D contributed to anti-tumor metastasis.

Specific inhibitors of PLD have been developed in recent years. There are several PLD1 and PLD2 specific inhibitors available in the market, including specific PLD1 inhibitors [21] (VU0155069 and VU-0359595), PLD2 specific inhibitors [22] (Halopemide, NOPT, and VU-0364739), and specific inhibitors of both PLD1 and PLD2 [23] (ML-299, VU-0155056, and VU-0285655-1). Among these inhibitors, 5-fluoro-2-indolyldes-chlorohalopemide (FIPI), firstly synthesized in 2007 [24], has been widely applied in anti-tumor growth and anti-metastasis via inhibiting PLD associated signal pathways [13, 14, 16, 25, 26]. However, free FIPI had pharmacokinetic defects, such as low bioavailability of 18% and high clearance rate [24], which required effective formulation to overcome its shortcomings and increase its biological activity. The combination with other drug and suitable formulations containing FIPI has not been reported yet.

Liposomes were widely applied in active and passive targeted drug delivery systems [27], and their advantages over other nanoparticles involved excellent biocompatibility such as protection against oxidative stress in an ex vivo human erythrocytes-based model [28] and no signs of necrosis or inflammation in normal tissues by histological examination [29] due to resemblance to biomembranes [30]. Liposomes can not only load active ingredients with various physicochemical properties, but also possess other optimized properties, such as avoiding the side effects of chemotherapy drug and keeping the entrapped therapeutic components from degradation [4, 31].

**Fig. 1:**
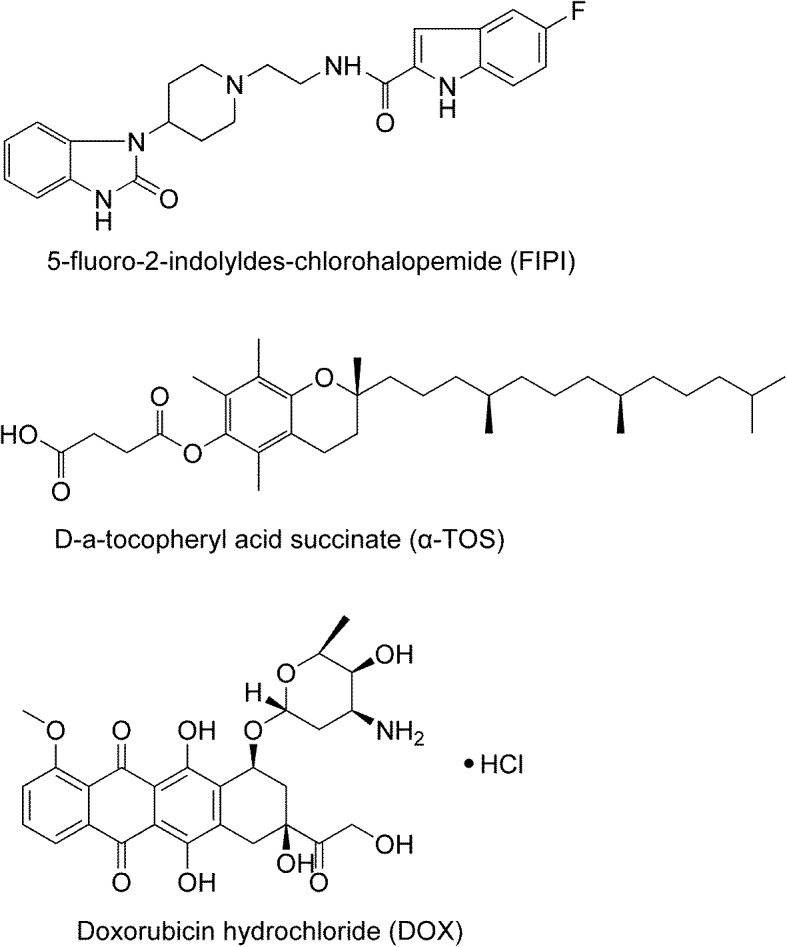
Structural formulas of 5-fluoro-2-indolyldes-chlorohalopemide (FIPI), D-α-tocopheryl acid succinate (α-TOS), and doxorubicin hydrochloride

The aim of this study was to construct the liposomes co-loading doxorubicin (DOX) as an anti-cancer model drug, FIPI as a specific PLD inhibitor, and D-alpha tocopheryl acid succinate (α-TOS) as a functional excipient that not only negatively charged the surface of the liposomes [32], but also exhibited several biological function such as induction of apoptosis, inhibition of cell proliferation, and P-gp ATPase [33, 34] in order to obtain the desired anti-tumor metastatic activity. Structural formulas of the above three components were shown in Fig. 1. A variety of liposomes were constructed using film dispersion method combined with the pH-gradient method. Characteristics of the liposomes such as size, potential, stability, and release profiles were explored. In vitro and in vivo studies, using highly metastatic breast cancer MDA-MB-231 cell line (MDA-MB-231) and MDA-MB-231 cell line that stably expressed luciferase (MDA-MB-231/Luc), were carried out to assess the efficacy of anti-metastasis and safety profiles of the liposomes.

## Materials and Methods

Egg phosphatidylcholine (EPC) was obtained from LIPOID (Germany). Cholesterol (Chol) was supplied from Sigma-Aldrich (St. Louis, MO, USA). Doxorubicin hydrochloride (DOX) and D- alpha-tocopheryl acid succinate (α-TOS) were obtained from Dalian Meilun Biotech Co., Ltd (Liaoning, China). 5-fluoro-2-indolyldes-chlorohalopemide (FIPI) was obtained from MedChem Express Co., Ltd (Shanghai, China). Sephadex G-25 and Sulforhodamine B (SRB) were from Shanghai Macklin Biochemical Co., Ltd (Shanghai, China). Matrigel was purchased from BD Biocoat (Franklin, NJ, USA). D-luciferin was bought from YEASEN Biotech Co., Ltd (Shanghai, China). Dulbecco’s modified Eagle’s medium (DMEM), paraformaldehyde (PFA), and 1% crystal violet were purchased from M&C GENE TECHNOLOGY LTD. (Beijing, China). Other reagents were analytical or high-performance liquid chromatography grade.

### Cell Culture and Animals

The human breast cancer MDA-MB-231 cell line was purchased from the Institute of Basic Medical Science, Chinese Academy of Medical Science (Beijing, China). Highly metastatic breast cancer MDA-MB-231 cell line that stably expressed luciferase (MDA-MB-231/Luc) was obtained from Peking University Medical and Healthy Analytical Center. Cells were maintained in Dulbecco’s modified Eagle’s medium (Macgene, Beijing, China) supplemented with 10% fetal bovine serum (FBS, Gibco, USA), 100 units/ml penicillin, and 100 μg/ml streptomycin at 37 °C in a humidified atmosphere with 5% CO_2_.

The female BALB/c nude mice (initial weight of 16–18 g) were purchased from the Peking University Experimental Animal Center (Beijing, China) and kept under SPF condition. All experimental procedures were performed in accordance with guidelines approved by the Ethics Committee of Peking University.

### Preparation of Liposomes

The liposome preparation was accomplished by film dispersion method and pH-gradient method [35]. Briefly, EPC, Chol, and α-TOS were co-dissolved in chloroform at a ratio of 20:5:2 (*w/w/w*) in a pear-shaped bottle and the solvent was evaporated completely by a rotary vacuum evaporator in a water bath at room temperature. The lipid film was hydrated with 300 mM citrate buffer (pH 2.50), which was shaken at 50 rpm for 3 min. The suspensions were subsequently sonicated with a probe sonicator for 15 min, and successively extruded for 3 times with a 0.22-μm polycarbonate membranes filter. Then, the liposomes were passed onto Sephadex G-25 gel column to exchange the outer aqueous solution for 10 mM PBS (pH7.40), which exhibited a transmembrane pH-gradient following gel filtration. To encapsulate FIPI into the liposome, FIPI dissolved in 300 mM citrate buffer was added into outer aqueous solution of liposome, pH of which was gradually adjusted to 7.40 with 1.0 M NaOH solution [36]. The liposome was incubated at 40 °C for 15 min in a water bath with magnetic stirring to encapsulate FIPI, and further incubated for 15 min after DOX dissolved in water was added into the liposome. After being separated on Sephadex G-25 gel column to remove the uncapsulated drug, the multifunctional liposomes (DFT-lip) were prepared. TOS liposomes (TOS-lip), FIPI liposomes (FIPI-lip), DOX liposomes (DOX-lip), DOX plus FIPI liposomes (DF-lip), and DOX plus TOS liposomes (DT-lip) were constructed using the same procedures by excluding the addition of DOX, FIPI, or α-TOS, respectively. Blank liposomes (Blank-lip) without α-TOS, FIPI, and DOX were prepared by film dispersion method as above.

### Characterization of Liposomes

The particle size, polydispersity index (PDI), and zeta potential values of liposomes were determined using Malvern Zetasizer Nano-ZS (Malvern, UK). The entrapped concentrations of DOX, FIPI, and α-TOS were assayed by HPLC. Chromatographic conditions of DOX and FIPI was as follows: column, Agilent Eclipse Plμs C18 column (5 μm, 4.6 × 250 mm); detection wavelength, 233 nm; column temperature, 25 °C; mobile phase, methanol, and sodium acetate buffer (30 mM, pH = 3.40) (55:45,*v*/*v*); flow rate, 1.0 ml/min. HPLC condition of α-TOS was as follows: column, Agilent Eclipse Plμs C18 column (5 μm, 4.6 × 250 mm); measuring wavelength, 285 nm; column temperature, 25 °C; mobile phase, methanol, and acetic acid (500:3.2, *v*/*v*); flow rate, 1.0 ml/min. The drug encapsulation efficacy (EE) and drug-loading content (LC) were calculated according to the following equations, respectively:

$$ \mathrm{EE}\%=\frac{\mathrm{amountofdrugencapsulated}}{\mathrm{amountofdrugused}}\times 100\% $$

$$ \mathrm{LC}\%=\frac{\mathrm{amout}\ \mathrm{of}\ \mathrm{drug}\ \mathrm{encapsulated}}{\mathrm{amount}\ \mathrm{of}\ \mathrm{drug}\ \mathrm{encapsualted}+\mathrm{amount}\ \mathrm{of}\ \mathrm{lipid}}\times 100\% $$

### In Vitro Release Behavior Study

The release of DOX and FIPI from DFT-lip in vitro was investigated by a dialysis method. Briefly, 1 ml of DFT-lip was added into the dialysis bag (MWCO 8000–14000 Da), which was immersed in 20 ml of release medium (10 mM PBS, pH7.4, pH5.0) and oscillated in a shaker (100 rpm) at 37 °C [37]. At different time point as designated, aliquots (1 ml) were withdrawn from the dialysate and replaced with equal volume of fresh PBS. The release amount of drug was determined by HPLC.

### Stability of Liposomes

The stability of DFT-lip at 4 °C and 25 °C was checked by Malvern Zetasizer Nano-ZS (Malvern, UK) [38]. The particle size and PDI of liposomes were measured after liposomes diluted with distilled water. The determination was conducted every three days during liposomes stored at 4 °C and 25 °C.

### Cellular Uptake

The cellular uptake characteristics of different liposomes and free DOX were evaluated by flow cytometry. MDA-MB-231 cells were seeded into 12-well plates at a density of 2 × 10^5^ cells/well and maintained for 24 h. Afterwards, the cells were exposed to medium without FBS (as control), DOX-lip, DFT-lip, and free DOX dissolved in PBS at a concentration of 5 μM DOX, respectively. After incubation for 4 h, the cells were harvested, washed with cold PBS, and resuspended in 500 μl cold PBS. The cells fluorescence was then detected using a flow cytometer (Becton Dickinson, USA). Each assay was repeated in triplicate.

### Cytotoxicity of Liposomes

The cell viability was tested by sulforhodamine-B staining assay [39] to investigate the effect of liposomal cytotoxicity on MDA-MB-231 cells status in wound healing, cell migration, and cell invasion assay. MDA-MB-231 cells were seeded into 96-well plates at a density of 8000 cells/well and incubated at 37 °C for 24 h. The cells were then treated with blank DMEM (as control), Blank-lip, FIPI-lip, DF-lip, DT-lip, and DFT-lip at a concentration of 2 μM FIPI for 7 h and 24 h, respectively. The cell culture supernatant was decanted and 200 μl of cold 10% (*w*/*v*) TCA was gently added to each well. After incubation at 4 °C for 1 h, the plates were washed five times with slow-running tap water and allowed plated to air-dry at room temperature. Each well was added into 100 μl of 0.4% SRB (*w*/*v*) solution and stained for 20 min at room temperature. Then, the plates were quickly rinsed five times with 1% (*v*/*v*) acetic acid to remove unbound dye and dried at room temperature. Finally, 150 μl of 10 mM Tris base solution (pH10.5) was added to each well, which was shook on a gyratory shaker for 30 min. The absorbance at 540 nm was measured by a microplate reader (Infinite F50, Tecan Group Ltd., Shanghai, China) and the viability of cells was calculated using the following formula:

$$ \mathrm{cell}\ \mathrm{viability}\left(\%\right)=\frac{\mathrm{absorbance}\ \mathrm{at}\ 540\ \mathrm{nm}\ \mathrm{for}\ \mathrm{treated}\ \mathrm{cell}\mathrm{s}}{\mathrm{absorbance}\ \mathrm{at}\ 540\ \mathrm{nm}\ \mathrm{for}\ \mathrm{control}\ \mathrm{cell}\mathrm{s}}\times 100\% $$

### Wound Healing Assay

MDA-MB-231 cells were firstly seeded in a 6-well culture plate at a density of 4 × 10^5^ cells/well and maintained in DMEM with 10% fetal bovine serum. After cells were grown to approximate 90% confluence, a scratch with constant width was done in monolayer of cells with a 200-μl pipette tip. The cells were washed twice with PBS to remove the suspended cells and further incubated for 48 h with blank DMEM (as control), Blank-lip, FIPI-lip, DF-lip, DT-lip, and DFT-lip at a concentration of 2 μM FIPI at 37 °C in a humidified atmosphere with 5% CO_2_, respectively. Wound closure was photographed at different time-points after scratch using a fluorescence microscope (Olympus, Japan).

### Cell Migration Assay and Cell Invasion Assay

MDA-MB-231 cells were serum-starved for 2 h and then resuspended at a density of 1.0 × 10^6^ cells/ml in serum-free DMEM. One hundred microliter of cell suspension were seeded into the upper chambers of transwell inserts (Corning, USA) that were separated from the lower wells by a 6.5-mm diameter and 8-mm-pore-size polycarbonate membrane [40]. The lower chambers were filled with 500 μl of DMEM with 10% FBS as a chemoattractant. Then, the cells were incubated with blank DMEM (as control) for 7 h, Blank-lip, FIPI-lip, DF-lip, DT-lip, DFT-lip at a concentration of 2 μM FIPI, at 37 °C in a humidified 5% CO_2_ cell culture incubator. After that, the cells on the upper side of the insert membrane were removed with cotton swabs. The cells on the lower side of the insert membrane were fixed with 4% paraformaldehyde (PFA) in phosphate-buffered saline (PBS) and stained with 1% crystal violet in methanol/PBS (1:4, *v*/*v*), and viewed under a microscope (Olympus, Japan).

Cell invasion assay was carried out similar to cell migration assay described above in the transwell chambers coated with Matrigel layer except for incubation time of 24 h.

### Prevention of Tumor Metastasis In Vivo and Safety Evaluation

To study the effects of liposomes on the prevention of tumor metastasis in vivo, an animal assay was performed following the method previously reported [41]. In brief, 200 μl of MDA-MB-231/Luc cell suspension at a density of 5 × 10^6^ cells/ml was inoculated via the tail vein of the nude mice, and 7 h after that, mice were treated with Blank-lip and DFT-lip intravenously at the dose of 2.0 mg FIPI/kg body weight every 2 days for 12 days, respectively. On day 14 after cell inoculation, the mice were starved for 24 h. After that, the substrate D-luciferin (150 mg/kg in Dulbecco’s phosphate-buffered saline (DPBS)) was intraperitoneally injected into the mice. Bioluminescence imaging was initiated 10 min after the injection with a total exposure time of 3 min, bin 8. Mice were constantly exposed to 3% isoflurane to sustain sedation during imaging. The signal intensity of lung metastasis was quantified as the sum of all detected photon counts within the region of interest (ROI). Identical illumination settings were used for acquiring all images.

For safety evaluation, the body weight of mice was monitored after every injection of preparation. In addition, on day 14 after cell inoculation, 20 μl of blood from the retro-orbital sinus of mice was collected and analyzed through the blood routine examination as a preliminary toxicity assessment of the treatment formulation [37].

### Statistical Analysis

The results were expressed as mean ± standard deviation. The SPSS 13.0 software (Chicago, Illinois, USA) was applied in the statistical analysis. An unpaired, two-tailed Student’s *t* test was used to determine the significance of the difference between two group means. Values of *p* < 0.05 meant statistically significant difference for all tests.

## Results

### Preparation and Characterization of Liposomes

Characterizations of the liposomes prepared were listed in Table 1. All of liposomes had an average particle size of about 84–120 nm with a narrow PDI ranged from 0.183 to 0.230, and were negatively charged. More specifically, the average diameter of liposomes containing one component, such as DOX, α-TOS, or FIPI, increased slightly to 84–110 nm as compared to that of Blank-lip (88.58 ± 0.27 nm). Similarly, the liposome particle size, which encapsulated two of them, varied in the range of 102–108 nm. In contrast, the DFT-lip loading all three components had the largest particle size, 119.00 ± 0.80 nm. In addition, the EE of liposomes encapsulated one component was over 94%, which was not remarkably different with those that encapsulated two or more components. In summary, all of the liposomes with small particle size, uniform particle size distribution, negative charge, and high EE, were prepared by the definite prescription and process, and the difference in the characteristics between different liposomes was not obvious.

**Table 1:** Characterization of all liposomes

Liposomes	Particle size	PDI	Zeta potential	Encapsulation efficiency(%)			Drug-loading content (‰)		
	(nm)		(mv)	DOX	FIPI	TOS	DOX	FIPI	TOS
Blank-lip	88.58 ± 0.27	0.230 ± 0.004	− 13.80 ± 0.66	–	–	–	–	–	-
DOX-lip	84.53 ± 0.45	0.225 ± 0.005	− 27.07 ± 0.38	96.83 ± 1.30	–	–	1.33 ± 0.01	–	-
FIPI-lip	93.58 ± 0.43	0.220 ± 0.017	− 31.63 ± 0.40	–	96.07 ± 1.85	–	–	8.76 ± 0.19	-
TOS-lip	108.60 ± 0.75	0.187 ± 0.007	− 50.33 ± 1.61	–	–	94.10 ± 0.03	–	–	66.92±0.67
DF-lip	102.73 ± 1.16	0.243 ± 0.009	− 27.97 ± 0.67	96.28 ± 2.50	94.80 ± 1.27	–	1.32 ± 0.03	8.86 ± 0.06	-
DT-lip	108.40 ± 0.87	0.183 ± 0.016	− 59.40 ± 1.22	94.58 ± 2.87	–	91.29 ± 2.84	1.19 ± 0.06	–	65.67±0.59
DFT-lip	119.00 ± 0.80	0.188 ± 0.010	− 58.50 ± 1.04	93.45 ± 3.71	91.46 ± 3.71	91.42 ± 1.94	1.19 ± 0.04	8.69 ± 0.19	65.16±0.18

All data presented here were calculated as the mean ± SD (*n* = 3) for three different preparations

### In Vitro Release

**Fig. 2:**
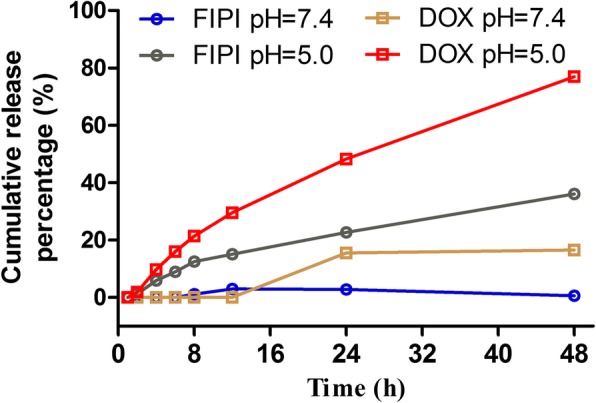
In vitro release profiles of FIPI and DOX from DFT-lip

As shown in Fig. 2, the in vitro release percentage of DOX and FIPI from the DFT-lip were below 2% within the initial 2 h at pH7.4 and pH5.0, indicating no burst release. Furthermore, the release of DOX and FIPI from the liposomes at pH7.4 was below 20% for 48 h, which meant little leakage outside liposomes into blood circulation.

### Shelf Stability of Liposomes

**Fig. 3:**
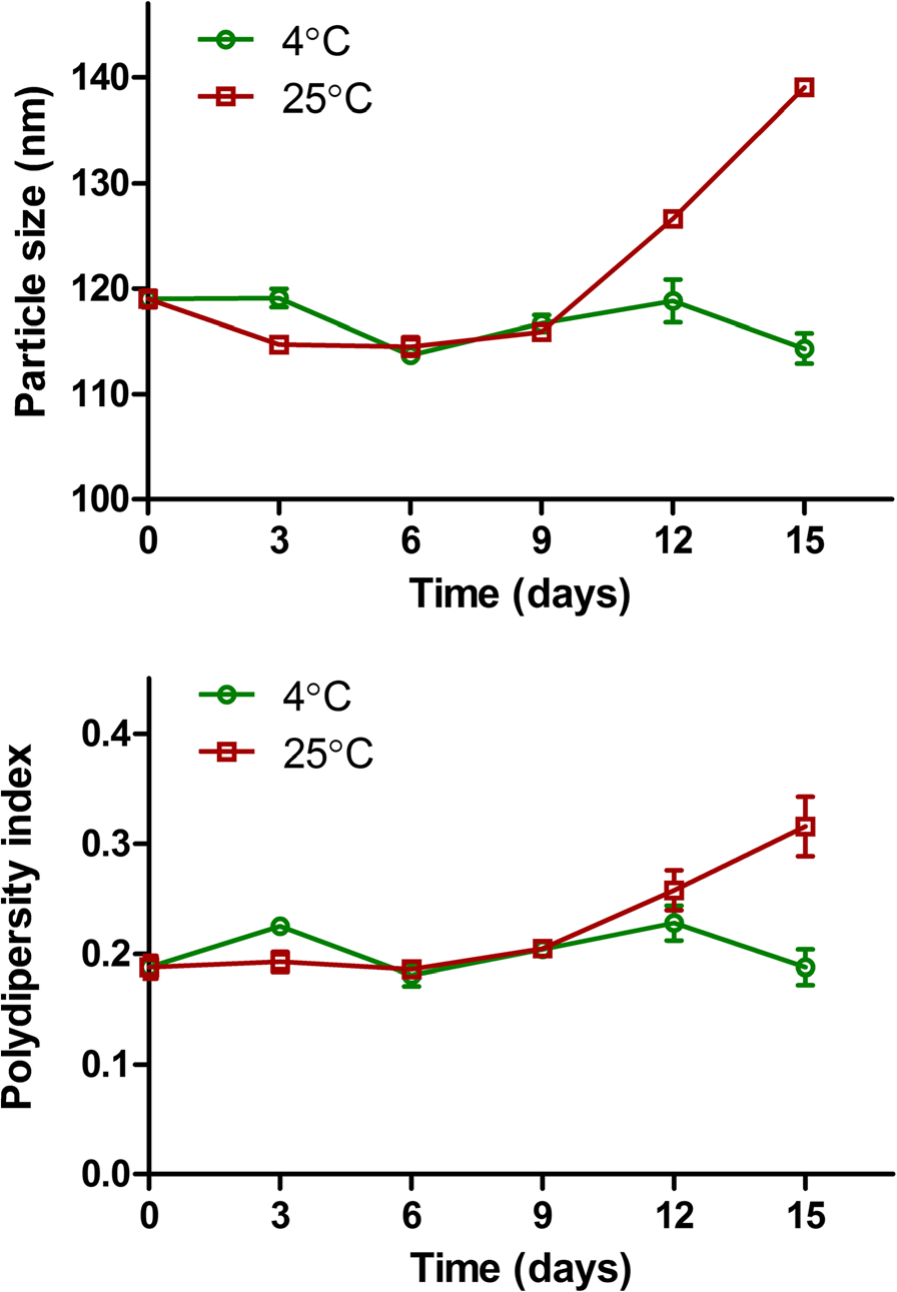
Stability of DFT-lip at 4 °C and 25 °C in PBS for 15 days determined by particle size and polydispersity index

The shelf stability of DFT-lip at different temperature was assessed by Malvern Zetasizer Nano-ZS. As shown in the Fig. 3, particle size and PDI of DFT-lip stored at 4 °C for 15 days and stored at 25 °C for 9 days were not altered obviously, while the remarkable increase in size and PDI was displayed for DFT-lip stored at 25 °C for more than 9 days. These stability data demonstrated that DFT-lip were stable at 4 °C for 15 days and at 25 °C for 9 days to reach the tumor by EPR effect.

### Cellular Uptake by MDA-MB-231 Cells

**Fig. 4:**
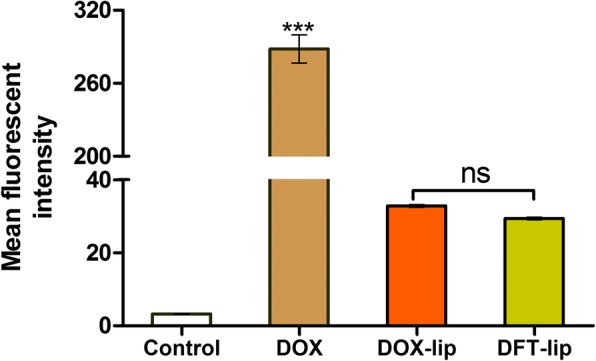
Flow cytometric measurement of cellular uptake by breast cancer MDA-MB-231 cells after incubation with free DOX, DOX-lip, and DFT-lip at a concentration of 5 μM DOX for 4 h at 37 °C. The auto-fluorescence of cells was applied as the control. All the data presented here were calculated as mean ± SD (*n* = 3). Notes: ns, *p* > 0.05; ***p < 0.001

From the flow cytometry analysis result as shown in Fig. 4, free DOX exhibited the highest fluorescent intensity than DOX-lip and DFT-lip (*p* < 0.001), indicating the highest cellular uptake. Compared to DOX-lip, the cellar uptake of DFT-lip was not significant (*p* > 0.05).

### Wound Healing, Cell Migration, and Invasion Assay

**Fig 5:**
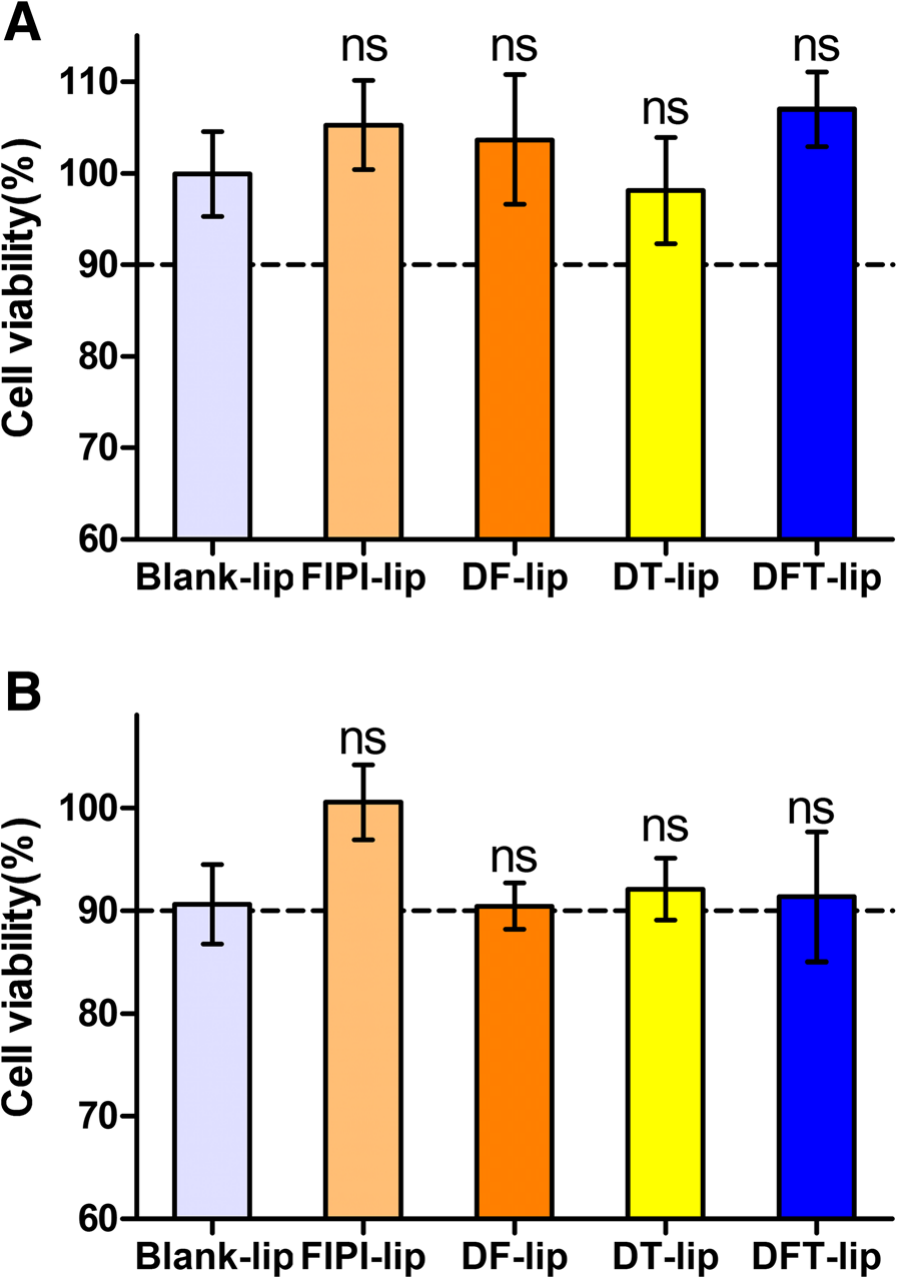
Cell viability of MDA-MB-231 cells incubated with Blank-lip, FIPI-lip, DF-lip, DT-lip, and DFT-lip for 7 h (**a**) or 24 h (**b**) at a concentration of 2 μM FIPI, respectively. All the data presented here were calculated as mean ± SD (*n* = 6). Notes: ns, *p* > 0.05 versus blank liposomes

As shown in Fig. 5, the viability of cells incubated at the same time and sample concentration as cell migration/invasion assay was above 90%, indicating that the inhibition ability on cell mobility, migration, and invasion was not caused by the cytotoxicity of the formulations. DOX, FIPI, and α-TOS were completely encapsulated within liposomes thereby avoiding false-positive results caused by its cytotoxicity.

**Fig. 6:**
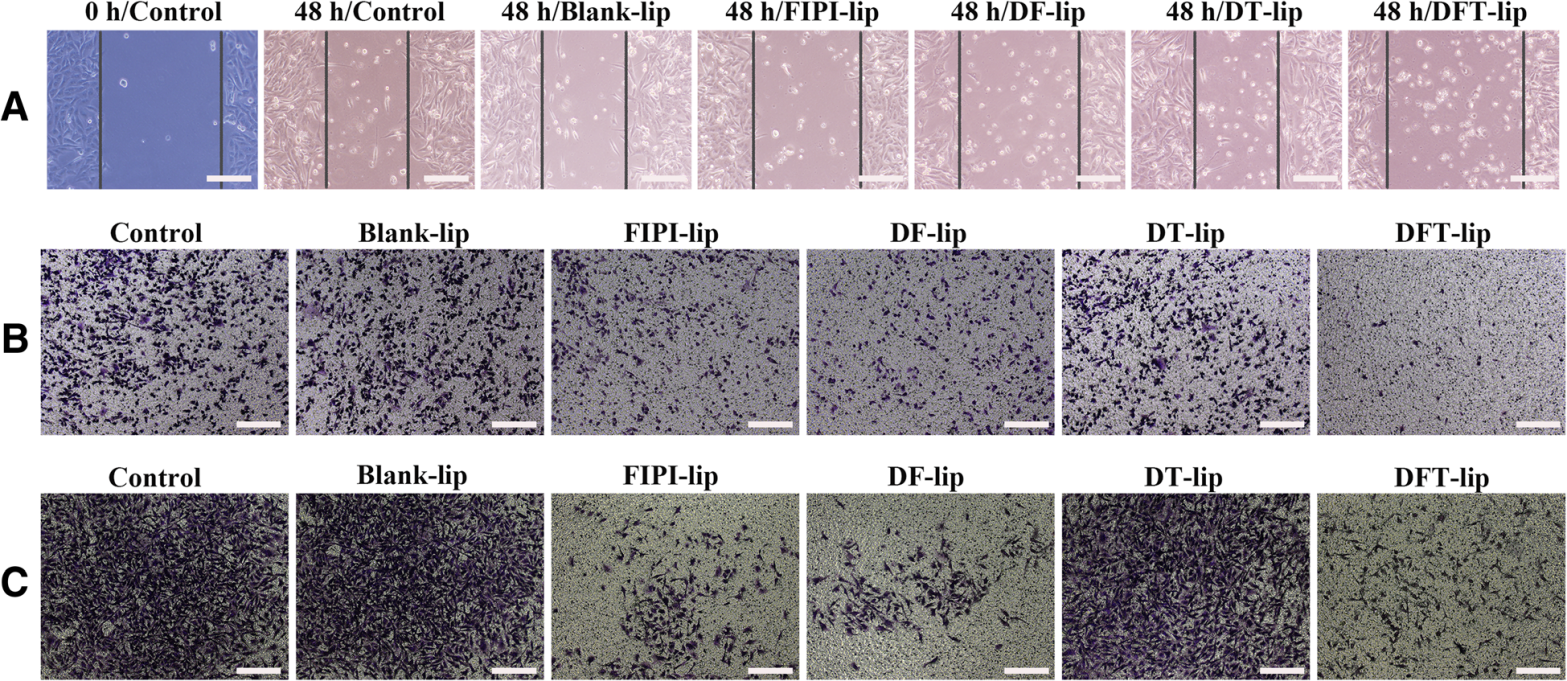
In vitro anti-metastasis study of the liposomes. **a** Inhibition effects on the cell motility in vitro. Images of MDA-MB-231 cells wound edge after incubation with control, Blank-lip, FIPI-lip, DF-lip, DT-lip, and DFT-lip for 48 h, respectively (magnification, × 100). **b** Inhibition effects on cell migration. Images of MDA-MB-231 cell migration after incubation with control, Blank-lip, FIPI-lip, DF-lip, DT-lip, and DFT-lip for 7 h at a concentration of 2 μM FIPI (magnification, × 100). **c** Inhibition effects on the cell invasion. Images of MDA-MB-231 cell invasion after incubation with control, Blank-lip, FIPI-lip, DF-lip, DT-lip, and DFT-lip for 24 h a concentration of 2 μM FIPI (magnification, × 100)

To investigate the effects of liposomes on cell motility, migration and invasion, wounding healing, cell migration, and invasion assays were conducted in MDA-MB-231 cells, respectively. As shown in Fig. 6a, the scratch in the control group was visibly healed after being incubated for 48 h, indicating excellent motility of MDA-MB-231 cells. Scratches in the Blank-lip and DT-lip group were invisible, while no obvious healing was observed in FIPI-lip, DF-lip, and DFT-lip group. It demonstrated that Blank-lip and DT-lip showed no inhibition ability on MDA-MB-231 cell mobility, whereas FIPI-lip, DF-lip, and DFT-lip could tremendously inhibit MDA-MB-231 cell mobility. Moreover, the result of the transwell migration and invasion assays also confirmed the effect of liposomes on cell migration inhibition. Cell migration and invasion assay were shown in Fig. 6b, c. There were more cells in the lower surface of the transwell chamber for the control, Blank-lip, and DT-lip group, indicating that liposomes without FIPI took no effect on cell migration/invasion. By comparison, less cells migrated/invaded to the lower surface of the transwell chamber in the FIPI-lip, DF-lip, and DFT-lip group, which illustrated that MDA-MB-231 cell migration or invasion was distinctly depressed by FIPI-lip, DF-lip, and DFT-lip. The results demonstrated that FIPI played a key factor in liposomal delivery system exerting anti-invasive and anti-migration.

### Prevention of Tumor Metastasis In Vivo

**Fig. 7:**
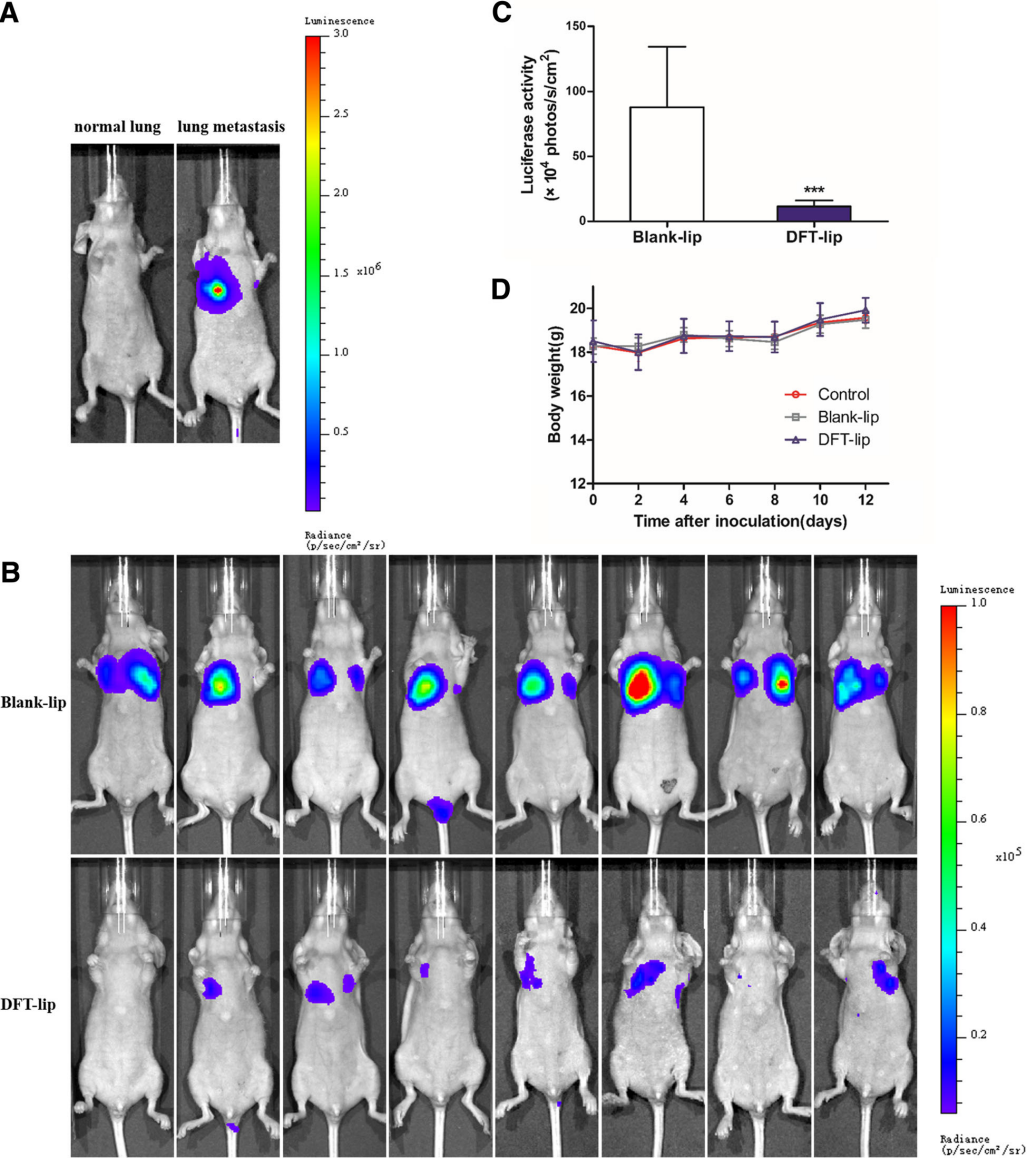
In vivo anti-metastasis study of the liposomes. **a** Bioluminescent images of the nude mice model of lung metastasis of breast cancer, which was constructed with the injection of MDA-MB-231/Luc cells via tail vein. **b** Representative in vivo bioluminescent images of mice in a preventive protocol treated with Blank-lip and DFT-lip (*n* = 8). **c** The extent of tumor metastasis burden was quantified (*n* = 8). Notes: ***p < 0.001 versus Blank-Lip. **d** Body weight changes for the tumor-bearing mice

As shown in Fig. 7a, the lung of normal nude mice was not fluorescent, while the nude mice injected with MDA-MB-231/Luc cells was fluorescent in the lung, demonstrating that the nude mice model of breast cancer metastasis was successfully constructed. As depicted in Fig. 7b, the lungs of nude mice in DFT-lip group showed weaker fluorescence than those in Blank-lip group, and two of nude mice in the DFT-lip group even did not exhibit fluorescence in the lung. The semi-quantitative result of fluorescence intensity as shown in Fig. 7c, there was a highly significant difference (*p* < 0.001) in the fluorescence intensities between the Blank-lip group and DFT-lip group. All data presented here indicated that DFT-lip could inhibit the progression and prevent the initiation of metastasis of highly metastatic breast cancer. In respect to the safety evaluation of liposomes in vivo, body weight monitoring of nude mice during treatment. As shown in Fig. 7d, the body weight of nude mice in each experimental group showed a similar trend that was an initially slight decrease and a subsequent rise. There was no significant difference (*p* > 0.05) among all the groups at different time points, indicating less systemic toxicity of Blank-lip and DFT-lip.

Besides, blood routine tests after treatment were conducted. Both Blank-lip and DFT-lip induced no significant changes (*p* > 0.05) in all blood routine results compared to the control group as listed in Table 2. In total, DFT-lip exhibited good safety profiles in vivo.

**Table 2:** Results of blood examination results from the retro-orbital sinus of mice after the administration of liposome formulations

Assay	Control	Blank-lip	DFT-lip
GR	1.25 ± 0.44	1.27 ± 0.51	1.09 ± 0.64
GR%	17.20 ± 5.44	17.29 ± 5.97	18.85 ± 12.29
HCT	49.04 ± 1.01	48.96 ± 2.24	48.41 ± 1.68
HGB	163.25 ± 4.27	160.00 ± 4.87	156.25 ± 5.34
LY	5.11 ± 0.96	4.43 ± 0.97	4.04 ± 1.07
LY%	69.81 ± 7.25	63.34 ± 8.49	63.99 ± 10.53
MCH	16.28 ± 0.38	16.28 ± 0.34	15.84 ± 0.35
MCHC	333.00 ± 9.21	327.13 ± 8.85	322.63 ± 6.21
MCV	48.85 ± 1.11	49.76 ± 1.26	49.10 ± 1.22
MO	0.94 ± 0.40	1.34 ± 0.43	1.09 ± 0.34
MO%	12.99 ± 5.56	19.37 ± 5.54	17.16 ± 3.74
MPV	5.31 ± 0.15	5.45 ± 0.21	5.51 ± 0.17
PCT	0.24 ± 0.03	0.26 ± 0.02	0.23 ± 0.03
PDW	13.61 ± 0.79	13.94 ± 0.96	13.74 ± 0.39
PLT	451.25 ± 47.78	481.75 ± 32.47	419.38 ± 44.64
RBC	10.04 ± 0.26	9.83 ± 0.42	9.85 ± 0.21
RDW	14.25 ± 0.97	14.19 ± 0.66	14.73 ± 1.07
WBC	7.30 ± 1.22	7.04 ± 1.29	6.21 ± 0.96

Data are presented as the mean ± SD (*n* = 8). *GR*, neutrophil granulocyte; *HCT*, hematocrit; *HGB*, hemoglobin; *LY*, lymphocyte; *MCH*, mean corpuscular hemoglobin; *MCHC*, mean corpuscular hemoglobin concentration; *MCV*, mean corpuscular volume; *MO*, intermediate cell; *MPV*, mean platelet volume; *PCT*, thrombocytocrit; *PDW*, platelet distribution width; *PLT*, platelet; *RBC*, red blood cells; *RDW*, red cell volume distribution width; *WBC*, white blood cells

## Discussion

Phospholipase D enzymes have long been proposed to play multiple cell biological roles in cancer progression, especially in metastasis [13, 14, 23]. Metastasis could be suppressed through inhibiting PLD activity by FIPI. In this study, multifunctional doxorubicin liposomes, containing FIPI, α-TOS, and DOX, were constructed for the inhibition of tumor metastasis.

At first, based on the different physicochemical properties of DOX, FIPI, and α-TOS, liposomes were prepared using film dispersion method, which encapsulated drug via pH-gradient method. α-TOS, a lipophilic, and amphiphilic ingredient was incorporated into the lipid bilayer when the lipid membrane was hydrated, while DOX and FIPI as weakly basic drugs penetrated the inner aqueous phase of liposomes through active pH-gradient encapsulation to achieve high drug loading. Since the transmembrane pH gradients (ΔpH) strongly influenced the equilibrium transbilayer distribution of weak bases across lipid membranes, the pH of the extraliposomal phase was precisely adjusted to achieve high encapsulation efficacy [42]. In addition, only in a dissolved state can the drug be actively encapsulated into the liposomes in response to ΔpH. In preparation of liposome drug loading, FIPI had a predicted solubility (25 °C) below 0.093 g/L at pH7.4, but approximately 3.5 g/L at pH2–4, calculated using Advanced Chemistry Development (ACD/Labs) Software V11.02, and was soluble in citrate buffer (pH2.5) but precipitated immediately in PBS (pH7.4) according to the pre-experimental results (data not shown). Therefore, the pH of the extra-liposomal phase should be lowered to dissolve sufficient FIPI and obtain the desired transmembrane loading of FIPI. However, this might induce the leakage of pre-encapsulated doxorubicin due to the decrease of the pH gradient. On the other hand, DOX powder or DOX aqueous solution could be directly added into the extraliposomal phase and be encapsulated into the liposomes without the decrease of ΔpH, which caused no leak of FIPI. Therefore, in order to achieve satisfactory co-encapsulation of FIPI with DOX, only FIPI was actively encapsulated firstly, following by DOX loading. The encapsulation efficiency of all the liposomes exceeded 94% as listed in Table 1, indicating that the above adjustment of liposomal preparation was reasonable and successful.

Except for high EE, the ideal liposomes should also demonstrate good characterizations of a low particle size, uniform size distribution, and a certain zeta potential. Among them, particle size is one of the most important parameters that determine the biological fate of carriers. Increased particle size reduces cell permeability [43] and cell uptake [44], alters tissue distribution characteristics [45], and is more easily recognized by the immune system to clear, thereby impairing passive targeting [46]. For the liposomes that encapsulated three ingredients (DOX, FIPI, and α-TOS), drug-loading process using a pH-gradient method caused a little increase in particle size to ~ 110 nm compared to that for the Blank liposomes, which tended to display high delivery efficiency and be accumulated into tumor tissues through permeability and retention (EPR) effect [47, 48]. The results demonstrated that the size of liposomes prepared using thin membrane together with pH-gradient method was not affected by more than two co-encapsulating drugs with different physicochemical properties. Zeta potential is another important parameter that influences the biological fate of particles that affected cellular adhesion/uptake and drug delivery [49]. Normally, liposomes with cationic lipids are prone to binding cells than liposomes with anionic lipids due to electrostatic interaction with negatively charged cell membrane (sialic acids and phospholipid head groups) [50]. DOX and FIPI with positive charge were encapsulated within the internal phase of the liposomes and therefore took no effect on the zeta potential of the liposomes. The carboxyl group of α-TOS located in the lipophilic interfacial region where it increased the membrane surface charge dissociated into negative ion at physiological pH, indicating that the zeta negative potential of the liposomes was further increased compared to that of the Blank liposomes. The enhanced electrostatic repulsion between particles helps to stabilize the liposomes during storage [51] and clinical application.

In the drug release study, pH5.0 and pH7.4 were used to simulate the physiological condition and endo-lysosomal environment of tumor cells. The release percentage of FIPI and DOX from the liposomes was higher at pH5.0 than that at pH7.4 as shown in Fig. 2, respectively. The relatively quick drug release rate at pH5.0 might be attributed to increased solubility of weakly basic DOX and FIPI in acidic release media. Therefore, after accumulated to tumor tissues via EPR effect and the liposomes were taken up by tumor cells via endocytosis and trapped in endosome. Its weakly acidic environment induced rapidly the release of drugs from liposomes in endosome to exhibit a therapeutic effect [47, 52].

It was generally believed that the uptake of drugs by cells was one of the key elements of efficacy. For the result that cellular uptake of liposomes loading doxorubicin was lower than free DOX, it was explained by the fact that free doxorubicin rapidly diffused into the cells [53], by contrast, liposomes enter the cell much slower by endocytosis. In our study, no significant difference was displayed in cellular uptake between DOX-lip and DFT-lip. This phenomenon was attributed to no obvious difference in particle size, zeta potential values, and no surface modification for different liposomes, due to the fact that particle size, zeta potential, and surface modification of nanoparticles influenced the cellular uptake [54–56]. The uptake results indicated that anti-invasion and migration effects might not be related to the uptake capacity of the liposomes.

**Fig. 8:**
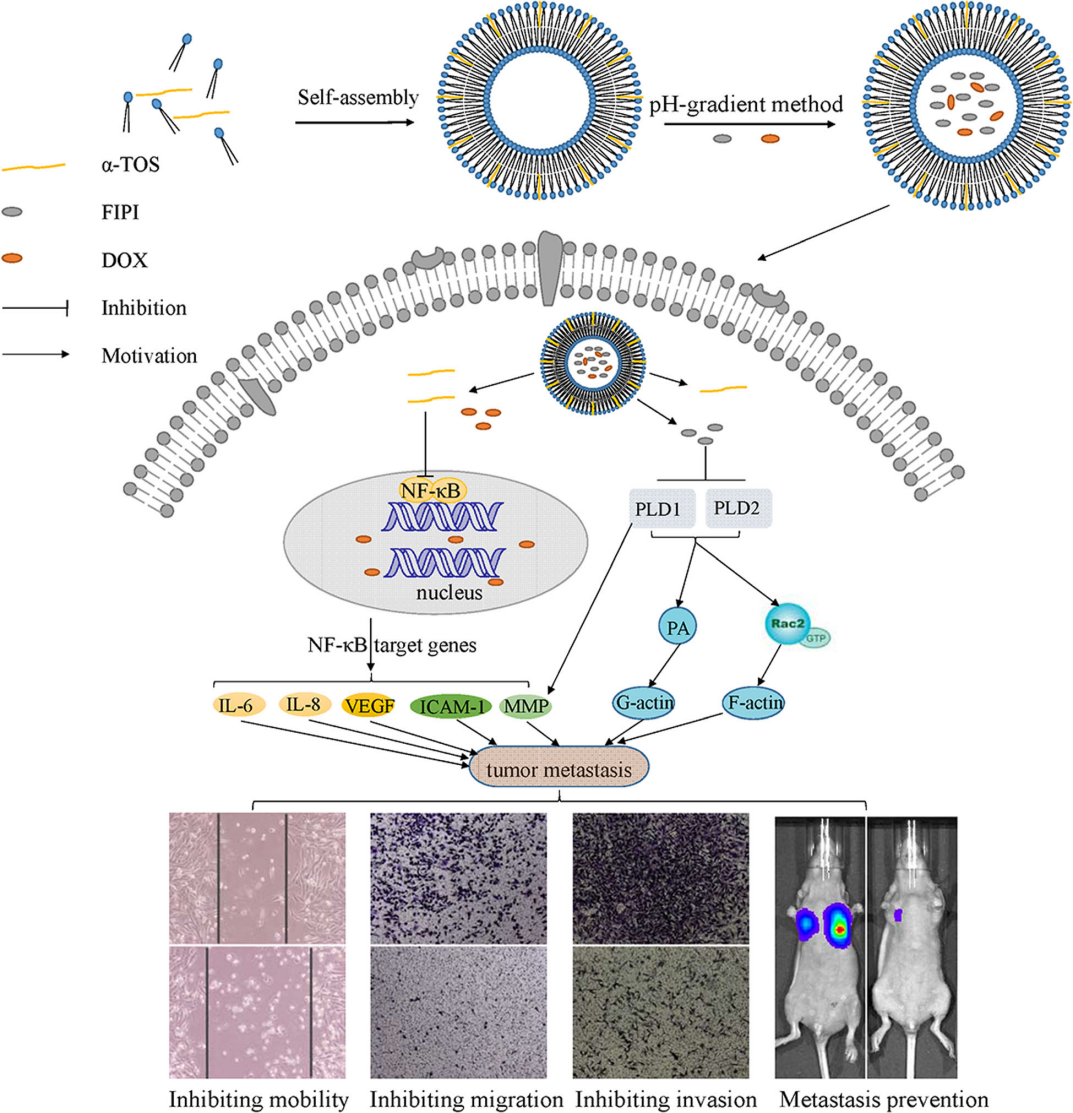
Schematic diagram of action mechanism for DFT-liposome

According to the results of wounding healing, cell migration and invasion assay, only the liposomes encapsulated FIPI apparently exhibited inhibitory effects on breast cancer MDA-MB-231cell mobility, migration, and invasion, and DT-lip showed no inhibitory effects in cell invasion assay, which contradicted the report that α-TOS could inhibit tumor cell invasion [57]. Doxorubicin at a non-toxic concentration induced cell migration and cell invasion in highly metastatic breast cancer cells [58, 59], thereby α-TOS alone could not offset the above effect of DOX without FIPI. More importantly, DFT-lip exhibited the strongest inhibitory effects among all the experimental groups, predicting a synergistic anti-metastatic effect between α-TOS and FIPI on metastatic potential MDA-MB-231cells. As already reported, α-TOS inhibited activity of NF-κB activity and reduced expression of IL-6, IL-8, and VEGF together with ICAM-1 [60], which was associated with the promotion of invasion and metastasis [61]. In addition, α-TOS inhibited cancer cell invasiveness associated with MMPs that were the key enzymes in the proteolysis of the basement membrane during invasion [57]. At the same time, PLD was downstream transcriptional target molecule of NF-κB; therefore, α-TOS might abolish PLD1 expression via inhibition of NF-κB transactivation [59], which assisted FIPI in synergistic anti-metastatic efficacy as illustrated with Fig. 8.

The metastatic process, from a physical point of view [62], can be termed a series of steps such as detachment, intravasation, circulation, extravasation, colonization, and eventually reactivation [5]. Once inside the circulatory system, the tumor cells aggregated through interaction with platelets and fibrinogen, shielding the tumor cells from being recognized and cleared by the immune system, promoting EMT and eventually assisting them in seeding at distal metastatic sites [63, 64]. In the above process, the NF-κB pathway, required for induction and maintenance of EMT, was also essential for extravasation and metastatic seeding [65]. Besides, the interaction between tumor cells and platelets was impaired in mice lacking PLD1 [26]. Therefore, the result of tumor metastasis in vivo demonstrated that DFT-lip not only suppressed EMT through α-TOS-mediated inhibition of NF-κB, but also interfered tumor cell-platelet interaction via lower PLD expression/activity suppressed by FIPI, preventing the initiation and progression of tumor metastasis.

## Conclusions

This study, for the first time, utilizes phospholipase D pathways to affect invasion, migration, or metastasis of tumor cells. Phospholipase D can promote tumor cell invasion and migration through multiple pathways. Therefore, inhibition of phospholipase D can obtain inhibitory effect from multiple pathways and is an efficient anti-invasion strategy.

In this study, three active ingredients (DOX, FIPI, and α-TOS) with different physicochemical properties were successfully co-loaded in liposomes that showed uniform particle size, high-encapsulation efficiency, negative charge, storage stability, and control release profiles to achieve co-delivery and ensure the efficacy of the three components as a promising anti-metastasis strategy.

## Abbreviations

Chol: Cholesterol
CSCs: Cancer stem cells
CTCs: Circulating tumor cells
CXCR-4: Chemokine receptor type 4
DMEM: Dulbecco’s modified Eagle’s medium
DOX: Doxorubicin
DPBS: Dulbecco’s phosphate buffered saline
DTCs: Disseminated tumor cells
EE: Encapsulation efficiency
EMT: Epithelial-mesenchymal transition
EPC: Egg phosphatidylcholine
FBS: Fetal bovine serum
FIPI: 5-fluoro-2-indolyldes-chlorohalopemide
GEF: Guanine nucleotide exchange factors
LC: Loading content
MET: Mesenchymal-epithelial transition
MMP: Matrix metalloproteinases
PA: Phosphatidic acid
PC: Phosphatidylcholine
PFA: Paraformaldehyde
PLD: Phospholipase D
SRB: Sulforhodamine B
TCA: Trichloroacetic acid
TGFβ: Transforming growth factor beta
TβRI-KI: Transforming growth factor beta type I receptor kinase inhibitors
α-TOS: D-α-tocopheryl acid succinate
